# Lack of Linkages among Fruiting Depth, Weight, and Maturity in Irrigated Truffle Fungi Marks the Complexity of Relationships among Morphogenetic Stages

**DOI:** 10.3390/jof7020102

**Published:** 2021-02-01

**Authors:** Sergi Garcia-Barreda, Sergio Sánchez, Pedro Marco, Gian Maria Niccolò Benucci, Vicente González

**Affiliations:** 1 Unidad de Recursos Forestales, Centro de Investigación y Tecnología Agroalimentaria de Aragón (CITA), Instituto Agroalimentario de Aragón—IA2 (CITA-Universidad de Zaragoza), Avda. Montañana 930, 50059 Zaragoza, Spain; ssanchezd@cita-aragon.es (S.S.); pmarcomo@cita-aragon.es (P.M.); 2 Centro de Investigación y Experimentación en Truficultura de la Diputación de Huesca (CIET), Polígono Fabardo s/n, 22430 Graus, Spain; 3 Department of Plants, Soil and Microbial Sciences, Michigan State University, East Lansing, MI 48824, USA; benucci@msu.edu; 4 Unidad de Protección Vegetal, Centro de Investigación y Tecnología Agroalimentaria de Aragón (CITA), Instituto Agroalimentario de Aragón—IA2 (CITA-Universidad de Zaragoza), Avda. Montañana 930, 50059 Zaragoza, Spain; vgonzalezg@aragon.es

**Keywords:** *Tuber melanosporum*, hypogeous fruitbodies, fruitbody formation, morphological traits, path analysis, truffle cultivation

## Abstract

The highly prized black truffle (*Tuber melanosporum*) has become a model species for ectomycorrhizal fungi biology. However, several questions concerning its reproductive phase remain unanswered. To provide new hypotheses on the fruitbody formation process, we have explored the causal links among development characters of black truffle fruitbodies that are primarily linked to either the mating process, fruitbody growing stage, or maturation. Path analysis was applied to test causal models outlining the relationships among fruitbody development characters such as fruiting depth, weight, shape, and spore maturity. These characters were investigated over a two-season survey and three soil typologies (plus peat-based substrate) under irrigated conditions. We found a clear and generalized relationship between fruitbody weight and shape. Among clusters of fruitbodies we found a positive relationship between the weight of the largest fruitbody and the weight of the remaining fruitbodies. However, no generalized relationships among characters linked to different development stages appeared. Our results were noticeably consistent across soil typologies, both for fruitbodies growing singly and in clusters, indicating that early-developing fruitbody characters did not influence characters linked to subsequent morphogenetic stages. The lack of links among stages opens new perspectives for pre-harvest quality management with stage-specific cultivation practices.

## 1. Introduction

The European black truffle (*Tuber melanosporum* Vittad., Pezizales) is an ectomycorrhizal fungus that in nature mostly grows in association with Angiosperm plants (e.g., *Fagaceae*). In cultivated orchards, the most common hosts are *Quercus* species. Truffle cultivation has advanced greatly in recent years, although it is not completely domesticated yet, and many biological and ecological aspects of the several processes involved still need clarification [1,2]. Black truffle has also attracted attention as a model ectomycorrhizal ascomycetous species for genomic studies, research on the mating process and population genetic structure, on fruitbody (FB) nutrition or on aroma [2,3,4,5]. Black truffle fruiting is a multigene-mediated process that follows specific and organized differentiation patterns and requires several months to reach completion [3,6,7]. The sequential morphogenetic stages leading to the FB formation can be classified into mating process, FB growing stage, and maturation [1,6,7,8]. However, very little is known about the intrinsic or environmental signaling pathways regulating truffle FB morphogenesis [1,7,9,10].

The mating process (from the stimulation of the formation of the mating structures to the mating itself) typically happens throughout late spring, apparently in several flushes [1] (Table 1). The precise location along the mycelial network where the mating event between mycelia of opposite mating types happens will determine the soil depth of the full-grown FB [2,11]. After the mating event, the FB starts to develop and its structure becomes gradually complex as the weight rapidly increases [7,8]. Growing below ground, the FB swelling and its final shape will be influenced by the soil mechanical constraints, with a relevant role of the characteristic pyramidal warts of the peridium [6]. At the end of the intense growth stage, the FB has practically achieved its final size. It is then, that the maturation stage begins, with the spores acquiring their characteristic pigmentation and the FB developing its unique aroma [7,12,13]. Maturation begins in late autumn, and the subsequent senescence processes set the moment in which dogs can localize the ripe FB. The FBs are harvested during several months throughout the winter, evidencing that the volatile compounds that attract dogs are not formed simultaneously in all FBs [5] (Table 1). Each one of the spots localized by a dog is excavated by the harvester: in most of the digs only one FB appears (single FBs), whereas in others, a cluster of FBs grow in very close proximity. Little scientific attention is usually paid to truffle FBs growing in clusters [14,15], although growing within these clusters could either affect FB formation patterns or could trade off with size due to localized resource depletion or inhibition mechanisms.

The outcome of the FB formation process relies upon how this sequence of stages (mating, growing and maturation) proceeds. The developmental patterns of FBs are affected not only by environmental but also by endogenous factors at different stages, such as the expression of certain enzymes related with melanin-synthesis pathways [10,16]. This raises the question of whether the moment and conditions in which a morphogenetic stage occurs might influence the following ones. Research on the relationships among FB development characters could help shed light on this aspect. As outlined above, a number of FB development characters that include fruiting depth, weight, shape, and spore maturity can be primarily linked to particular morphogenetic stages (Table 1). Since these characters define or influence the commercial quality standards of truffle FBs [17], understanding the relationships among development characters and the processes that shape them may also open new perspectives for pre-harvest quality management through improved farming practices.

Here, we aimed to: (i) build a causal model to explain how development characters of truffle FB influence one another, and (ii) test whether these relationships are consistent across different soils and dig typologies (single FBs and FB clusters). We tested several alternative models for each dig typology in three replicate blocks along a soil texture gradient that is representative of common truffle orchard soils, and compared these mineral soils with the FBs growing within a peat-based substrate amendment. The causal models were built considering the linkages between the studied characters and the sequential morphogenetic stages of truffle FBs: fruiting depth linked to the mating process, weight and shape linked to the growing stage, and spore maturity linked to maturation [1,7,8] (Table 1). We hypothesized that: (i) fruiting depth would have a positive effect on weight and maturity, because soil depth buffers extreme values in temperature and water content, which are particularly variable in Mediterranean climates [18,19]; (ii) no relationship between weight and maturity would appear, because dogs usually localize full-developed ripe FBs of sizes from less than 10 g to more than 100 g; (iii) shape of small FBs would we more rounded, because they need to make and occupy less soil volume and are less likely to face mechanical constraints during growth; (iv) in FB clusters, the weight of the largest FB would show a negative relationship with the weight of the remaining FBs, due to the local resource depletion or inhibition mechanisms hypothesized by Moore et al. [20]; and (v) differences among soils and with substrate would affect relationships among FB development characters, since soil properties and localized substrate amendments are able to influence these characters [21].

## 2. Materials and Methods

### 2.1. Experimental Site

The study was conducted in a 15-ha truffle orchard established in 2001 with *Quercus ilex* subsp. *ballota* and *Quercus faginea* seedlings (arranged in rows 2:1) inoculated with *T. melanosporum* [21]. The experimental site is located in Gúdar-Javalambre county (Teruel province, eastern Spain, 1150 m a. s. l.). The climate is Continental Mediterranean, with a mean annual rainfall of 519 mm and a mean annual temperature of 11.1 °C, typical of Spanish truffle-producing regions [22]. The experimental site is placed in the piedmont of Gúdar mountain range, with calcareous soils developed on Cretacic clayey limestone in the upper part (block 3) and on Tertiary siltstones/sandstones in the lower part (block 1) (Table S1). In all the blocks, the 0–30 cm soil horizon in which almost all truffles grow is a homogeneous plow layer created after repeated tillage operations (during truffle cultivation and previously during many decades of cereal cultivation).

Truffles are harvested by the owner once a week throughout the fruiting season (November to March). Each year, when the fruiting season is over, the soil shallowly tilled, and a peat-based substrate is applied in ten spots around each tree [21]. The orchard is irrigated with a sprinkling system from April to October during the dry periods with scarce rainfall.

Peat-based amendment is aimed at increasing fruiting depth and shape of FBs [21]. In the experimental site, the peat-based amendment is being applied by the grower following the most common procedure used in Spain. The localized application of peat-based substrate around the host trees involves digging tronconical holes about 25 cm deep, filling them with about 1.5 L of a European *Sphagnum* peat-based substrate (Turbatruf^®^ from Projar, Quart de Poblet, Spain): A black peat—white peat—coir—perlite mix 11–5–3–1, with pH raised to 7.5) and re-covering the substrate with soil [21]. Grinded ripe truffle FBs are mixed with the substrate before being incorporated into the soil. Annually, part of the FBs grow in the bulk soil whilst another part appear within the substrate spots.

### 2.2. Experimental Design and Data Collection

In the experimental site, three replicate blocks of 0.25 ha with different soil textures were selected in a soil gradient along a 300-m-length transect line, with block 1 having sandy loam texture, block 2 having loam texture and block 3 having loam/clay loam texture (Table S1). Although in the wild black truffle is found in almost every type of texture, this texture gradient is representative of the range of common soil textures in black truffle cultivation [23]. The three blocks were managed with the same agronomic practices.

During the 2016–2017 and the 2017–2018 fruiting seasons each block was surveyed seven times from November to March. A total of 604 single FBs and 308 FB clusters were measured after being systematically localized and harvested by the grower with the aid of trained dogs (Tables S2 and S3). Fruiting depth was recorded as the depth in the soil of the bottom part of the deepest FB in the dig, at 10 cm intervals. The shape was evaluated as a combination of sphericity (ratio between measured diameters), and visually-estimated lobularity (percent surface occupied by lobules) and average height of lobules (in relation to FB size). This resulted in a shape index with nine categories, with higher values indicating higher-priced round, regular shapes (Table S4). A spore maturity index was calculated as the proportion (from 0 to 1) of asci containing mature (i.e., dark brown) spores, but this index is only available for single FBs. For each FB, a hymenial sample reaching 5–10 mm under the peridium was taken with a scalpel, and a minimum of 50 randomly selected asci were counted in each sample under light microscope, following Zeppa et al. [24]. Fresh weight was measured to the nearest 0.1 g after gently removing soil and substrate with a brush. Weight was measured in every FB in season 2017–2018, whereas for 2016–2017 only the weight of single FBs is available. The research dataset is available as Supplementary File S2.

The weight of FBs growing in clusters during season 2016–2017 was estimated through a partial least squares regression model fitted with the complete dataset from season 2017–2018 (*n* = 1047). This model was fitted with seven components, mainly based on FB maximum and minimum diameter. It accounted for 97% of the variability in the FB weight of season 2017–2018 (Table S5, Figure S1). It was validated with the available 2016–2017 measurements (single FBs, *n* = 275). The regression between log-transformed predicted and actual values of season 2016–2017 was highly significant (*p* < 0.001) and presented a R^2^ value of 0.96.

### 2.3. Statistical Analysis

The causal relationships among FB development characters were evaluated using the d-sep method of path analysis [25], with the aid of the R package ggm [26]. Path analysis has been applied to study causal patterns between morphological, physiological, and ecological attributes in plant biology and agronomy [25,27]. The d-sep method judges if a particular model is consistent with the experimental data. For each model, it involves: (i) specifying a causal hypothesis in the form of a directed acyclic graph, (ii) identifying the set of independence claims (basis set) implied in the model, (iii) calculating the null probability associated with each claim, (iv) combining these probabilities using Fisher’s C statistic, and (v) comparing this C with the fixed significance level [25]. If a path model exhibited a *p*-value for Fisher’s C higher than 0.05, it was considered consistent with the data [25]. When more than one path model was consistent with the data, they were compared with the Akaike’s Information Criterion corrected for small sample size, AIC_c_ [28].

We separately analyzed single FBs and FB clusters, to assess whether the strength and pattern of the relationships among development characters was consistent between both dig typologies. Since peat shows distinctive and unique features in comparison with mineral soils [29]—that provoking differences in truffle fruiting depth, FB weight, shape and occurrence of clusters [21]—FBs growing within the peat-based substrate across the three blocks were grouped and analyzed separately from mineral soils. The bulk soil of each replicate block (BS1, BS2 and BS3) was analyzed separately to assess whether the nature of the relationships among characters was general across soils, since the weight and shape of FBs can be influenced by soil properties [21]. Since our study is not aimed at characterizing year-to-year variability, FBs from both sampled fruiting seasons were combined.

For single FBs, we built three alternative path models to test the relationships among the day of the season in which the FB was harvested (harvesting date, HD), fruiting depth, weight, shape and spore maturity. The three alternative models assumed a relationship between weight and shape, as well as an effect of HD on weight and maturity, which are widely accepted by growers and researchers. Model A assumed that characters linked to a particular morphogenetic stage are not influenced by those linked to previous stages (Table 1). Model B assumed that weight is influenced by fruiting depth, and that spore maturity is influenced by fruiting depth and weight. Model C assumed that weight and shape are influenced by fruiting depth, and that maturity is influenced by fruiting depth and weight (Figure S2).

The three models were compared following the d-sep method outlined above. Once selected a best-fit model, each of its constituent paths was modelled with generalized additive models, in order to allow for non-linear relationships and different types of error distribution [30]. A Poisson error distribution was used for fruiting depth and shape, assessing the model fit through overdispersion. A Gaussian (normal) distribution was used for weight and maturity. In these models, the assumptions of normal distribution and constant variance were assessed, with weight being log-transformed to more closely meet the assumptions. The analyses were conducted with the R package mgcv [31,32].

For each path in the best-fit model, we present the *p*-value, the shape of the estimated relationship and the percent deviance explained by each variable, calculated as the reduction in deviance after dropping that term while maintaining the same smoothing parameters throughout. The latter is aimed at comparing the relative contribution of each variable, because we avoided standardization to keep the relationships between characters with its original shape.

For FB clusters, we built five alternative path models to test the relationships among HD, fruiting depth, weight and shape of the largest FB in the cluster, and combined weight of all the other FBs in the cluster (Figure S3). Maturity was not included due to data unavailability. All the models assumed a weight-shape relationship for the largest FB, as well as an effect of HD on weight. Models A–C assumed that characters of the largest FB are not influenced by the weight of the remaining FBs, whereas models D and E assumed that the weight and shape of the largest FB are influenced by the weight of the remaining FBs. Models A and D assumed that neither weight nor shape is influenced by fruiting depth (which is linked to a previous stage); models B and E assumed that weight is influenced by fruiting depth; and model C assumed that both weight and shape are influenced by fruiting depth. When analyzing the best-fit path models, a Gamma error distribution was used for the weight of the remaining FBs.

## 3. Results

### 3.1. Single Fruitbodies

**Figure 1:**
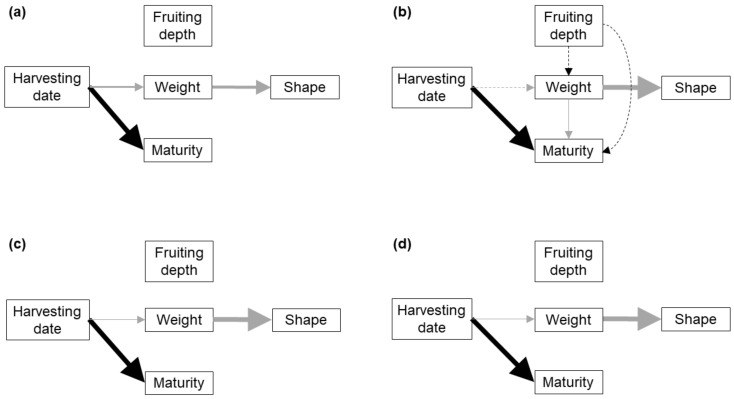
Best-fit path models showing the causal links among development characters in FBs growing singly in peat-based substrate (**a**), and the bulk soil of block 1 (**b**), block 2 (**c**) and block 3 (**d**). Solid lines indicate significant links between the variables, dashed lines indicate non-significant links included in the model, black lines indicate positive relationships and grey lines negative relationships. The thickness of an arrow is proportional to the percentage of deviance explained by a particular variable.

The three path models proposed (Figure S2) were consistent with the collected data for the peat-based substrate amendment (hereafter called substrate) and the bulk soil of the three replicate blocks (*p* > 0.05, Table 2). However, in the substrate, BS2 and BS3, model A reached a much lower AIC_c_ value and a much higher weight, indicating that, according to Shipley [28] criterion, model A allowed a much better fit to the data (Table 2). In BS1, models B and C presented similar AIC_c_ values, much lower than that of model A (Table 2). However, only model B is shown as the best-fitting model because the equation parameters are very similar, and in both cases the paths linking fruiting depth with other variables are not statistically significant (Figure 1).

**Figure 2:**
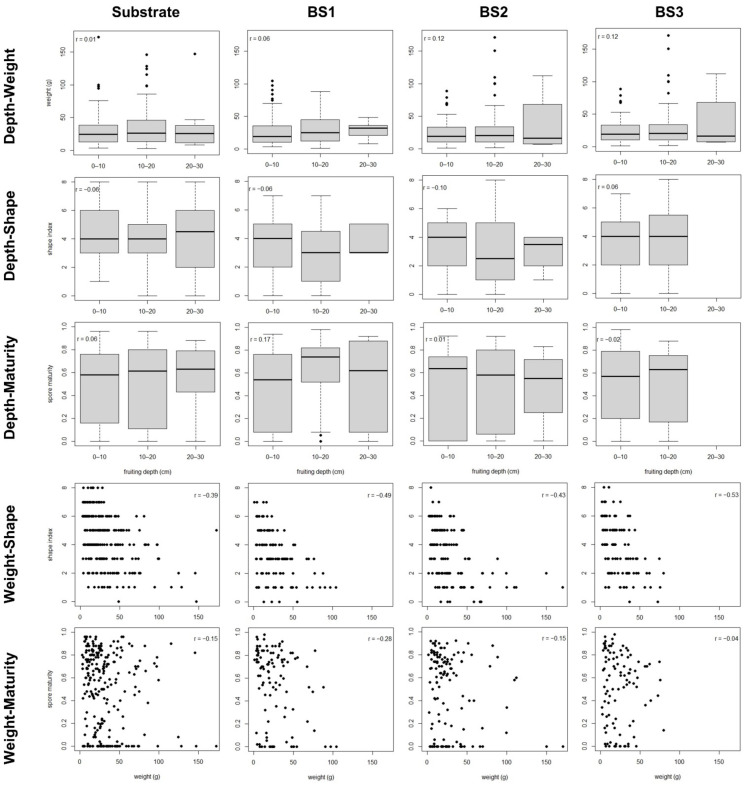
Scatter plot data for the development characters in the single fruitbodies of the studied blocks. Pearson’s correlation coefficient is reported for each bivariate relationship. BS1–BS3: bulk soil of blocks 1–3.

The best-fit path models for each soil typology (Figure 1) shared the following features: (i) fruiting depth did not show a significant relationship with any other character, (ii) FB weight showed a strong negative relationship with the shape index (i.e., bigger FBs having more irregular, less rounded shapes; Table S4), and (iii) the HD showed a strong positive relationship with spore maturity (Table 3 and Table 4, Figures S4–S7). The HD showed a significant and negative relationship with FB weight in the substrate, BS2 and BS3, but no significant relationship in BS1. Fruitbody weight showed a significant and negative relationship with maturity in BS1, which was not found in any other soil (Table 3 and Table 4; Figures S4–S7). The same associations between development characters were observed in the bivariate analyses (Figure 2).

The best-fit path models did not explain more than 6% of the variability in FB weight in any soil typology, while they explained 16–29% of the variability in the shape index and 23–41% of the variability in the spore maturity (Table 3 and Table 4). In these best-fit path models, the variability in FB weight was exclusively explained by HD, whereas the variability in shape was explained by the weight and the variability in spore maturity was mainly explained by HD, with weight also contributing to explain the variability of maturity in BS1 (Table 3 and Table 4). The relationship between weight and shape was negative, with clear differences between FBs smaller than 25 g and FBs larger than 50 g (Figures S4–S7). The relationship between weight and maturity in BS1 was negative but plateauing above 10 g, corresponding to a mean FB diameter of 2.5–3 cm (Figure S5).

### 3.2. Fruitbody Clusters

Among the five alternative path models proposed (Figure S3), model D was the one that reached lower AICc value and higher weight for all the analyzed soil typologies, indicating that, according to Shipley [28] criterion, model D allowed the best fit to the data (Table 5).

**Figure 3:**
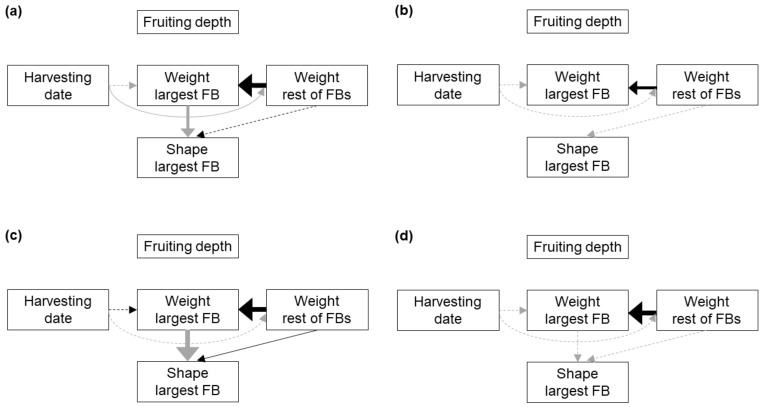
Best-fit path models showing the causal links among development characters in FBs growing in clusters in peat-based substrate (**a**), and the bulk soil of block 1 (**b**), block 2 (**c**) and block 3 (**d**). Solid lines indicate significant links between the variables, dashed lines indicate non-significant links included in the model, black lines indicate positive relationships and grey lines negative relationships. The thickness of an arrow is proportional to the percentage of deviance explained by a particular variable. FB: fruitbody.

**Figure 4:**
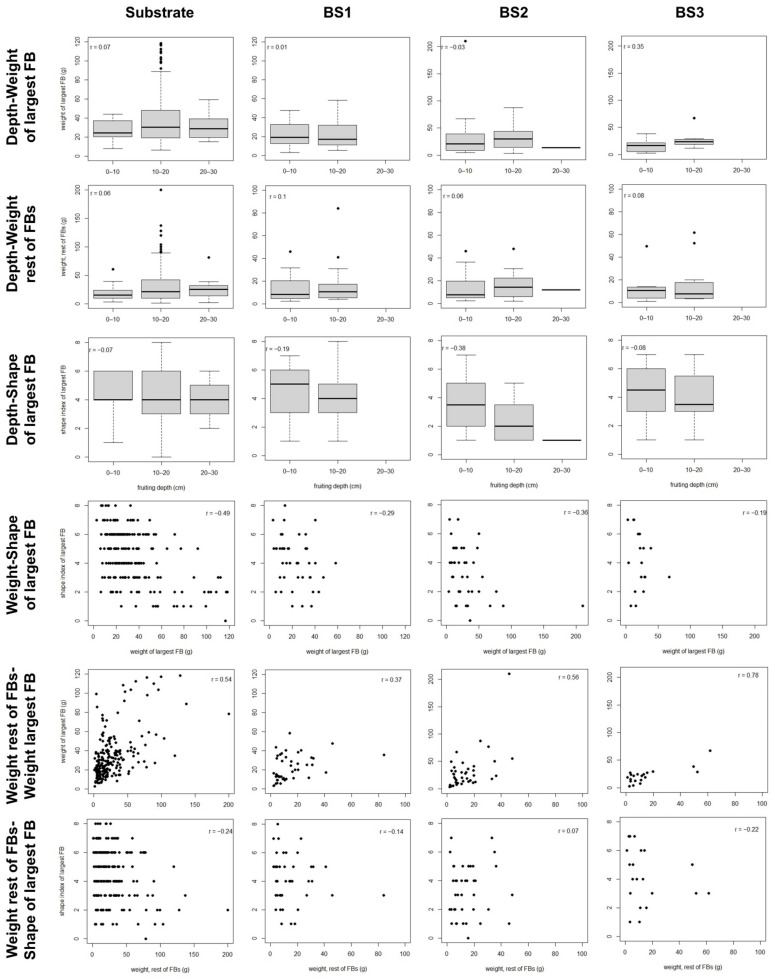
Scatter plot data for the development characters in the fruitbodies growing in clusters in the studied blocks. Pearson’s correlation coefficient is reported for each bivariate relationship. BS1–BS3: bulk soil of blocks 1–3.

The best-fit path models for each soil typology (Figure 3) shared the following features: (i) fruiting depth did not show a significant relationship with any other character, (ii) the weight of the largest FB in a cluster showed a strong positive relationship with the combined weight of all the other FBs in the cluster, and (iii) the HD did not show a significant relationship with the weight of the largest FB in the dig (Table 6, Figures S8–S11). In the substrate and BS2, the weight of the largest FB in the cluster showed a strong negative relationship with its shape index (Table 6, Figures S8 and S10). In the substrate the HD showed a significant and negative relationship with the weight of the remaining FBs in the cluster (Table 6; Figure S8). Finally, in BS2 the shape index of the largest FB of the cluster showed a significant and positive relationship with the weight of the remaining FBs (Table 6; Figure S10). The same associations between development characters are suggested by the bivariate analyses, although in some cases concealed by the fact that other variables as the HD are also involved in the relationship (Figure 4).

The best-fit path models explained 19–29% of the variability in the weight of the largest FB in the cluster, while they explained less than 4% of the variability in the weight of the remaining FBs (Table 6). For the shape index of the largest FB, the best-fit models in substrate and BS2 explained 18–23% of the variability, whereas in BS1 and BS3 none of the analyzed variables showed a significant relationship with shape (Table 6). In the best-fit models, the variability in the weight of the largest FB was exclusively explained by the weight of the remaining FBs, whereas the weight of the remaining FBs was only affected by HD (Table 6). The relationship between the weights of the largest FB and the remaining ones was in all cases positive (Figures S8–S11). Finally, the variability in the shape index of the largest FB was mainly explained by its weight, with the weight of the remaining FBs also contributing to explain the variability of shape in BS2 (Table 6). The relationship between weight and shape was negative, with clear differences between FBs smaller than 25 g and FBs larger than 50 g (Figures S8 and S10). In BS1 and BS3, in which no significant effect of weight on shape was found, the upper range value for the weight was 59–67 g, much lower than in the remaining soil typologies (Table S7). In BS2, the relationship between the shape of the larger FB and the weight of the remaining FBs was positive, with shape showing larger variability when the weight of the remaining FBs was high (Figure S10).

## 4. Discussion

The soil depth at which truffle FBs grew did not show any causal link with FB weight, shape, or spore maturity for any of the soil or dig typologies analyzed. This suggests that, under the experimental conditions, weight and maturity do not depend on FB location throughout the soil profile. This was unexpected, because it is generally accepted that soil depth determines FB sensitivity to bioclimatic damages such as those caused by drought or heat waves [33]. It is generally accepted that soil temperature and water regime follow marked depth gradients, with more extreme values and more rapid fluctuations near the soil surface [18,19]. Montant and Kulifaj [9] experimentally found that increasing soil temperature during winter and spring made truffle FBs grow earlier—as indicated by the soil surface cracking linked to the swelling of shallow FBs- and ripen earlier—as indicated by the harvesting date [34]. For other hypogeous ectomycorrhizal fungi, Luoma [35] and Luoma et al. [36] found a negative influence of thick layers of organic matter on fruiting, assuming that it was due to poor CO_2_ diffusion. However, in our experimental site the existing ranges in microclimatic variables did not trigger changes in weight or maturity throughout the soil profile for any soil typology. Despite this, microclimatic gradients in the soil play a role in the mating process of black truffle and thus on productivity of truffle orchards [1].

The growth of the truffle FB seems primarily related to the uptake, metabolism, and translocation of carbon from the plant host, and to nitrogen and water balances between fungus and host [4,37,38], although a hypothetical genetic influence on weight could also play a role. Besides, for many fungal species good aeration is associated with successful growth of FBs, in relation to gas exchange with the surrounding soil and to intensification of oxidative metabolism [20]. Thus, the soil depth at which the truffle grows and its weight seem to be determined by a different set of edaphoclimatic factors, with the host plant playing a more relevant role in the case of weight. The existence of differential gene expression patterns for each development stage is also relevant, and gene expression can also be influenced by environmental factors. Montanini et al. [39] hypothesized that the abundance of up-regulated transcription factors in *T. melanosporum* FB indicates a high degree of functional specialization in the reproductive stage. Moreover, Hacquard et al. [37] underscored the variety of metabolic pathways reflecting complex genetic processes during the truffle FB formation.

The causal relationship between weight and shape, both linked to the intense growth stage, was clear and general among all the soil and dig typologies, with the exception of BS1 clusters being likely related to a narrower range in weight. The existence of a negative relationship between FB weight and shape index (i.e., bigger FBs showing more irregular, less rounded shapes) is generally accepted between growers and researchers. From early in the development (peridial stage) the peridium warts are formed to help the FB swell [6,7], and the mechanical interaction with the surrounding soil matrix (including stones and roots) models the FB shape as it grows. Smaller FBs need to make and occupy less soil volume and are less likely to face mechanical constraints during growth. However, the fact that in all soil typologies the nine shape categories were present marks the great variability of this character (Tables S6 and S7) and of related mechanical constraints at relatively low spatial scales.

No causal link between FB maturity and weight was found, except for single FBs in BS1. Black truffle growers generally accept that weight and maturity are not related within the usual range of weight for full-developed healthy FBs. We found no evidence that the FBs must reach a minimum size to ripen (or that weight increase and maturation evolve together) for the range of weights studied, with minimum weights of 1–3 g in all soil typologies (Tables S6 and S7). This size is already attained by some FBs during the veined stage, typically happening in July–August [7]. Regarding the exception found in BS1, a negative relationship restricted to the smallest FBs was found in this block. This could be related to the fact that spore samples were taken near the peridium. In our experience, during early season it is not uncommon that spore maturity shows a certain degree of heterogeneity within a FB. The negative relationship could be explained by the fact that spore maturity could be more homogeneous in the hymenium of small FBs and our sampling overestimated the differences between small and large FBs, in relation to the fact that the development and maturation of the spores in a FB is asynchronous and progresses from the center of fertile veins outward and from the center of the gleba outward [6]. This underlines how important it might be for future truffle research to develop unbiased indicators and non-destructive sampling methods for assessing spore maturity [40].

We found no evidence that the maturation stage of the truffle FB is influenced by its weight, whereas previous research pointed that it is regulated through a decrease in tyrosinase activity [12,16]. The transition from the growing to the maturation stage could also be regulated by environmental cues, as suggested by the fact that November-December temperatures influence the mean date of truffle harvesting [41]. Several genes coding for photoreceptors and light-dependent regulators have been found in *T. melanosporum*, suggesting that seasonal variations in light, temperature, and/or oxygen concentration could mediate FB morphogenesis [3]. However, *T. melanosporum* apparently lacks regulators of circadian rhythmicity, which is a common indicator of seasons in plants [3].

Finally, a positive relationship between the weight of the largest FB and the weight of the remaining FBs was clear among the FB clusters of all the analysed soils. This was not expected. We had initially hypothesized that local resource depletion would trigger compensating mechanisms between FB survival and FB size in a dig, in consonance with the fact that, before summer, the density of *T. melanosporum* FBs (still immature) in the soil is much higher than the density of ripe FBs localized by dogs in the fruiting season [1,42]. The positive relationship we found could be explained if the growth of the largest FB was enhanced by the remaining FBs loosening the surrounding soil. However, the relationship between these development characters is very strong in the substrate, which is much looser than the bulk soil.

Alternatively, the positive relationship between the weights of the FBs in a dig could be related to local conditions in the soil microenvironment or to carbon transfer from the host. For other fungi, Moore et al. [20] posited that once a fungal genet begins to fruit the distribution of FBs is controlled by a flow of resources towards particular FBs rather than by local depletion of nutrients or by inhibition mechanisms. For hypogeous fungal communities in natural forests, Hunt and Trappe [43] and Luoma [35] found that, at a small plot level (4 m^2^), the relationship between total biomass and number of FBs was positive and not plateauing (although with low R^2^), either for the whole hypogeous community or for particular species. The hypothetical genetic influence on weight could play a role, considering that FBs in a cluster are likely to share maternal genetic material [2].

Overall, our results were noticeably consistent among the studied soil typologies, despite the fact that environmental gradients linked to soil depth and soil typologies influence the mating, FB survival, weight and number of FBs per dig [1,21]. The relationships among FB characters linked to different stages were, in general, not significant or of low magnitude. The lack of causal links among subsequent stages could be generalized for *T. melanosporum*, with the existence of morphogenetic stage switches governing which environmental or physiological cues play a role in each stage. Alternatively, the lack of causal links among stages could be specific to low-stress conditions for FB formation. Cultural practices (e.g., irrigation, soil tillage, and peat amendments) reduce the impact of stressful, limiting factors (e.g., plant competition for light, rhizosphere diversity, nutrients or space, summer aridity or high soil resistance to penetration). In herbaceous crops, compensating mechanisms among yield-related traits are typically higher in strong-stress than in low-stress conditions [27]. It would be interesting to test whether our results can be generalized to high-stress environments such as rainfed orchards or wild truffle stands. The genetic structure of *T. melanosporum* population in the experimental site could also play a role, considering that truffle orchards usually show high genetic diversity due to nursery and field practices [2].

Our approach did not allow to identify which specific environmental or physiological conditions influence development characters of FBs. Among the environmental factors potentially involved, not only abiotic factors but also microbial communities could play a critical role. The nature of the relationships between a fungus and the associated microbial communities is complex, ranging from defense against competition to modulation of physiological or developmental processes [44,45]. Kues and Liu [46] suggested that in certain saprophytic taxa, such as cultivated *Agaricus* spp., the presence of pseudomonads was essential for fruiting events. In other cases, some microorganisms influence the fruiting process by eliminating inhibitory compounds [47]. In this regard, truffle FBs and mycorrhizae harbor a poorly-understood microflora that could play a role in the mating, growing or maturation [48,49,50]. Further research on transcriptional gene expression throughout the distinct morphogenetic stages would greatly help identify the environmental and/or physiological factors governing each phase [7]. This would also help understanding how this genetic program is switched on, whether it is expressed constitutively or cumulatively, and the intensity with which the fungus perceives the different environmental inputs that model these morphogenetic processes.

## 5. Conclusions

To conclude, we found a clear and generalized relationship between FB weight and shape, with smaller FBs having less irregular, more rounded shapes. In FB clusters, we found an unexpected positive relationship between the weight of the largest FB and the weight of the remaining FB of the cluster. However, no generalized relationships among characters linked to different development stages appeared, thus indicating that under irrigated conditions, the factors that influence early-developing characters do not influence indirectly characters linked to subsequent stages. This suggests a scenario in which the development characters associated with a specific morphogenetic stage are independent from the conditions in which the previous stages occur. In this scenario, the development of certain FB characters is triggered by the interaction between a differential genetic expression program and a set of bioclimatic, edaphic, or biotic factors. Moreover, these factors are probably influencing the development characters of FBs with different intensity, specific weight or sensing mode, depending upon each morphogenetic phase of the FB. The lack of generalized trade-offs among subsequent morphogenetic stages opens new perspectives for pre-harvest quality management with stage-specific cultivation practices (e.g., devising practices aimed at increasing FB weight taking into consideration only factors influencing the intense growth stage, regardless of the fruiting depth). However, for this, it would be necessary a better understanding of the environmental factors that influence FB growth and maturation timing.

## Figures and Tables

**Table 1:** Relations between morphogenetic stages and fruitbody development characters (based on Zarivi et al. [7]).

Stage of Fruitbody Morphogenesis	Associated Development Characters	Period
Formation of mating structures (gametes), mating, and early stage of fruitbody differentiation (hyphal stage)	Fruiting depth	May–June
Fruitbody growth: development and swelling (peridial, veined, ascal and sporal stages)	Weight, shape	July–early November
Maturation (pigmented stage). Ripening (aroma development)	Spore maturity, harvesting date	November–March

**Table 2:** Model fit of the three competing path models (Figure S2) for fruitbodies growing singly in the peat-based substrate and in the bulk soil of each block. Bold letters indicate the models selected according to the model weight (C: Fischer’s C statistic, df: degrees of freedom, P: null probability, K: number of parameters needed to fit the model, AICc: Akaike value, W: model weight).

Model	C (df, P)	K	AICc	W
Substrate				
**A**	**12.5 (14, 0.56)**	**11.6**	**36.9**	**0.80**
B	9.7 (8, 0.28)	14.5	40.7	0.12
C	8.2 (6, 0.23)	15.5	41.4	0.08
Bulk soil of block 1				
A	22.3 (14, 0.07)	10.5	45.6	0.05
**B**	**9.9 (8, 0.27)**	**13.5**	**40.8**	**0.50**
C	7.5 (6, 0.28)	14.5	41.1	0.45
Bulk soil of block 2				
**A**	**7.9 (14, 0.90)**	**10.6**	**31.0**	**0.83**
B	5.2 (8, 0.74)	13.6	35.6	0.08
C	3.3 (6, 0.77)	14.3	35.7	0.08
Bulk soil of block 3				
**A**	**10.9 (14, 0.69)**	**10.5**	**34.6**	**0.89**
B	9.0 (8, 0.35)	13.5	40.3	0.05
C	6.7 (6, 0.35)	14.2	40.1	0.06

**Table 3:** Null probability (P) and percent deviance explained (D^2^) for each path in the best-fit model for fruitbodies growing singly in substrate and the bulk soil of Soil blocks 2 (BS2) and 3 (BS3).

Response	Predictor	Substrate		BS2		BS3	
		P	D^2^	P	D^2^	P	D^2^
Weight ^1^	Harvesting date	<0.001	5.8	0.03	3.6	0.04	4.1
Shape	Weight	<0.001	16.4	<0.001	21.9	<0.001	28.7
Maturity	Harvesting date	<0.001	40.8	<0.001	35.5	<0.001	34.2

^1^ Variable log-transformed.

**Table 4:** Null probability (P) and percent deviance explained by each variable (D^2^) for each path in the best-fit model for fruitbodies growing singly in the bulk soil of block 1.

Response	Predictor	P	D^2^
Weight ^1^	Harvesting date	0.13	-
	Fruiting depth	0.20	-
Shape	Weight	<0.001	23.6
Maturity	Harvesting date	<0.001	22.7
	Weight	0.01	4.2
	Fruiting depth	0.47	-

^1^ Variable log-transformed.

**Table 5:** Model fit of the five competing path models (Figure S3) for fruitbody clusters in the substrate and in the bulk soil of each block. Bold letters indicate the models selected according to the model weight (C: Fischer’s C statistic, df: degrees of freedom, P: null probability, K: number of parameters needed to fit the model, AICc: Akaike value, W: model weight).

Model	C (df, P)	K	AICc	W
Substrate				
A	74.9 (14, <0.001)	-	-	-
B	60.0 (10, <0.001)	-	-	-
C	61.7 (8, <0.001)	-	-	-
**D**	**6.6 (10, 0.76)**	**13.3**	**35.2**	**0.85**
E	2.6 (6, 0.86)	16.4	38.6	0.15
Bulk soil of block 1				
A	22.3 (14, 0.07)	8.4	43.6	0.11
B	18.0 (10, 0.06)	10.5	46.1	0.03
C	17.1 (8, 0.03)	-	-	-
**D**	**10.4 (10, 0.41)**	**10.8**	**39.7**	**0.81**
E	7.4 (6, 0.29)	13.2	45.8	0.04
Bulk soil of block 2				
A	29.4 (14, 0.009)	-	-	-
B	26.7 (10, 0.003)	-	-	-
C	20.7 (8, 0.008)	-	-	-
**D**	**7.6 (10, 0.67)**	**11.2**	**39.9**	**0.99**
E	6.9 (6, 0.33)	13.7	50.0	0.01
Bulk soil of block 3				
A	19.3 (14, 0.15)	8.2	87.7	<0.01
B	12.7 (10, 0.24)	10.2	59.4	0.39
C	12.2 (8, 0.14)	11.2	70.1	<0.01
**D**	**9.5 (10, 0.49)**	**10.5**	**58.6**	**0.60**
E	0.8 (6, 0.99)	12.6	78.9	<0.01

**Table 6:** Null probability (P) and percent deviance explained by each variable (D^2^) for each path in the best-fit model for fruitbody clusters in substrate and the bulk soil of blocks 1 (BS1), 2 (BS2) and 3 (BS3) (We.largest: weight of the largest fruitbody in the dig, We.rest: combined weight of all the other fruitbodies in the dig).

Response	Predictor	Substrate		BS1		BS2		BS3	
		P	D^2^	P	D^2^	P	D^2^	P	D^2^
We.rest	Harvesting date	0.04	3.2	0.80	-	0.054	-	0.50	-
We.largest	Harvesting date	0.72	-	0.053	-	0.38	-	0.19	-
	We.rest	<0.001	26.1	0.004	18.9	<0.001	29.2	0.014	28.6
Shape	We.largest	<0.001	18.3	0.13	-	0.002	23.0	0.70	-
	We.rest	0.93	-	0.92	-	0.049	2.2	0.58	-

## Data Availability

The data presented in this study are available in File S2 (Supplementary Materials).

## Supplementary Materials

The following are available online at https://www.mdpi.com/2309-608X/7/2/102/s1, Table S1: Physicochemical properties of bulk soil in the three blocks of the study site. Table S2: Number of single fruitbodies sampled, according to harvesting date. Table S3: Number of fruitbody clusters sampled, according to harvesting date. Table S4: Categories defined for the estimation of fruitbody shape index. Table S5: Loadings of the PLS regression to predict fruitbody weight. Table S6: Descriptive statistics for the characters of single fruitbodies. Table S7: Descriptive statistics for the characters of fruitbody clusters. Figure S1: Actual weight vs. weight predicted by the PLS regression. Figure S2: Path models tested for single fruitbodies. Figure S3: Path models tested for fruitbody clusters. Figure S4: Predicted relations among characters in single fruitbodies in the substrate. Figure S5: Predicted relations among characters in single fruitbodies in the bulk soil of block 1. Figure S6: Predicted relations among characters in single fruitbodies in the bulk soil of block 2. Figure S7: Predicted relations among characters in single fruitbodies in the bulk soil of block 3. Figure S8: Predicted relations among characters in fruitbody clusters in the substrate. Figure S9: Predicted relations among characters in fruitbody clusters in the bulk soil of block 1. Figure S10: Predicted relations among characters in fruitbody clusters in the bulk soil of block 2. Figure S11: Predicted relations among characters in fruitbody clusters in the bulk soil of block 3. File S2: Research dataset.
