# Supplementary material for: Lack of Linkages among Fruiting Depth, Weight, and Maturity in Irrigated Truffle Fungi Marks the Complexity of Relationships among Morphogenetic Stages
Source: J Fungi (Basel). 2021 Feb 1;7(2):102. doi: 10.3390/jof7020102 (PMC7912816; doi:10.3390/jof7020102)
Supplement: Supplementary file 1 [file jof-07-00102-s001.zip › File1_supplementary_material.pdf]

Supplementary material

Lack of linkages among fruiting depth, weight, and maturity in irrigated truffle fungi marks the complexity of relationships among morphogenetic stages

Sergi Garcia-Barreda, Sergio Sánchez, Pedro Marco, Gian Maria Niccolò Benucci, Vicente González

Table S1 Physicochemical properties of bulk soil in the three blocks of the study site
Table S2 Number of single fruitbodies sampled, according to harvesting date
Table S3 Number of fruitbody clusters sampled, according to harvesting date
Table S4 Categories defined for the estimation of fruitbody shape index
Table S5 Loadings of the PLS regression to predict fruitbody weight
Fig. S1 Actual weight vs. weight predicted by the PLS regression
Fig. S2 Path models tested for single fruitbodies
Fig. S3 Path models tested for fruitbody clusters
Fig. S4 Predicted relations among characters in single fruitbodies in the substrate
Fig. S5 Predicted relations among characters in single fruitbodies in the bulk soil of block 1
Fig. S6 Predicted relations among characters in single fruitbodies in the bulk soil of block 2
Fig. S7 Predicted relations among characters in single fruitbodies in the bulk soil of block 3
Fig. S8 Predicted relations among characters in fruitbody clusters in the substrate
Fig. S9 Predicted relations among characters in fruitbody clusters in the bulk soil of block 1
Fig. S10 Predicted relations among characters in fruitbody clusters in the bulk soil of block 2
Fig. S11 Predicted relations among characters in fruitbody clusters in the bulk soil of block 3
Table S6 Descriptive statistics for the characters of single fruitbodies
Table S7 Descriptive statistics for the characters of fruitbody clusters

Table S1 Soil physiochemical properties of the bulk soil from the three blocks (BS1, BS2, and BS3) in the study site. Soils were analysed at the *Laboratorio Agroambiental* (Government of Aragón).

	BS1	BS2	BS3
Sand (%)	54.1	46.4	31.7
Silt (%)	27.3	34.5	44.4
Clay (%)	18.6	19.1	23.9
pH, in water 1:2.5	8.3	8.5	8.5
Conductivity (dS m^{-1})	0.4	0.2	0.2
Organic matter (%)	1.2	1.2	1.5
Nitrogen, Kjeldahl (%)	0.07	0.07	0.09
C : N ratio	10.8	9.9	10.0
Phosphorus, Olsen (mg kg^{-1})	5	4	4
Potassium, ammonium acetate extracted (mg kg^{-1})	118	202	246
Calcium carbonate (%)	48	44	51
Active limestone (%)	9.0	6.6	10.5
Magnesium, ammonium acetate extracted (mg kg^{-1})	182	242	314

Table S2 Number of single fruitbodies sampled, according to the harvesting date (HD). Data are disaggregated for the peat-based substrate of the study site and for the bulk soil of each block (BS1, BS2 and BS3) (HD = 0 for the first day of the official harvesting season in Teruel province –15 November– and HD = 120 for the last day –15 March).

HD	-13	1	3	20	27	35	56	77	84	97	112	127	Total
Substrate	11	15	10	21	15	28	33	27	22	27	33	10	252
BS1	10	7	0	7	7	8	17	8	10	10	20	10	114
BS2	7	10	10	9	11	10	18	10	10	10	20	10	135
BS3	7	8	0	10	1	9	18	10	10	10	10	10	103

Table S3 Number of fruitbody clusters sampled, according to the harvesting date (HD). Data are disaggregated for the peat-based substrate of the study site and for the bulk soil of each block (BS1, BS2 and BS3) (HD = 0 for the first day of the official harvesting season –November 15th– and HD = 120 for the last day –March 15th).

HD	-13	1	3	20	27	35	56	77	84	97	112	127	Total
Substrate	9	26	4	19	23	21	40	18	16	14	8	5	203
BS1	8	4	0	3	0	3	7	6	5	2	2	5	45
BS2	2	3	4	2	6	4	7	2	1	4	5	0	40
BS3	0	2	0	2	0	5	3	2	3	3	0	0	20

Table S4 Categories defined for each one of the parameters used to estimate the fruitbody shape index (Dmin: minimum diameter, Dmax: maximum diameter). The shape index was calculated as the sum of the values assigned to sphericity, lobularity and height of lobules, thus ranging between 0 and 8.

Parameter	Description
Sphericity	Ratio between the measured Dmin and Dmax
3	Dmin : Dmax ratio higher than 0,85
2	Dmin : Dmax ratio between 0.7 and 0.85
1	Dmin : Dmax ratio between 0.5 and 0.7
0	Dmin : Dmax ratio lower than 0.5
Lobularity	Percentage of the fruitbody surface occupied by lobules, visual estim.
3	No lobules
2	Lobules occupying 0-25% of the peridium surface
1	Lobules occupying 25-50% of the peridium surface
0	Lobules occupying more than 50% of the peridium surface
Height of lobules	Average height of lobules in relation to fruitbody size, visual estim.
2	No lobules
1	Short lobules
0	Tall lobules (for fruitbodies of about 10 g, lobules taller than 4 mm with respect to the theoretical ellipsoid defined by Dmax and Dmin; for fruitbodies of about 30 g, lobules taller than 6 mm; and for fruitbodies of more than 70 g, lobules taller than 10 mm)

Table S5 Loadings (and squared weights) of the predictor variables for each of the seven components retained in the partial least square regression performed to predict fruitbody weight (Dmax: maximum dimeter, Dmin: minimum diameter, HD: harvesting date). For each component the cumulative percentage of variance of the response variable explained by the model (R^2-response) is included.

Predictor	PC1	PC2	PC3	PC4	PC5	PC6	PC7
Dmax							0.518 (0.29)
Dmin							0.25 (0.09)
$Dmax^2$			0.77 (0.57)	-0.61 (0.31)	0.21 (0.04)		
$Dmin^2$			0.28 (0.08)	0.37 (0.02)	-0.96 (0.82)	0.27 (0.05)	
Dmax × Dmin			0.43 (0.21)	0.75 (0.66)	0.29 (0.06)	-0.23 (0.03)	
$Dmax^2$ × Dmin	0.84 (0.70)	-0.60 (0.27)	0.19 (0.03)				
Dmax × $Dmin^2$	0.54 (0.30)	0.90 (0.59)	-0.32 (0.10)				
HD						-1.50 (0.36)	0.77 (0.59)
No order within the dig [1]							-0.312 (0.03)
R^2-response	62.34	62.88	91.52	92.85	92.93	92.95	97.32

[1] The fruitbodies within a dig were hierarchically ordered according to their weight

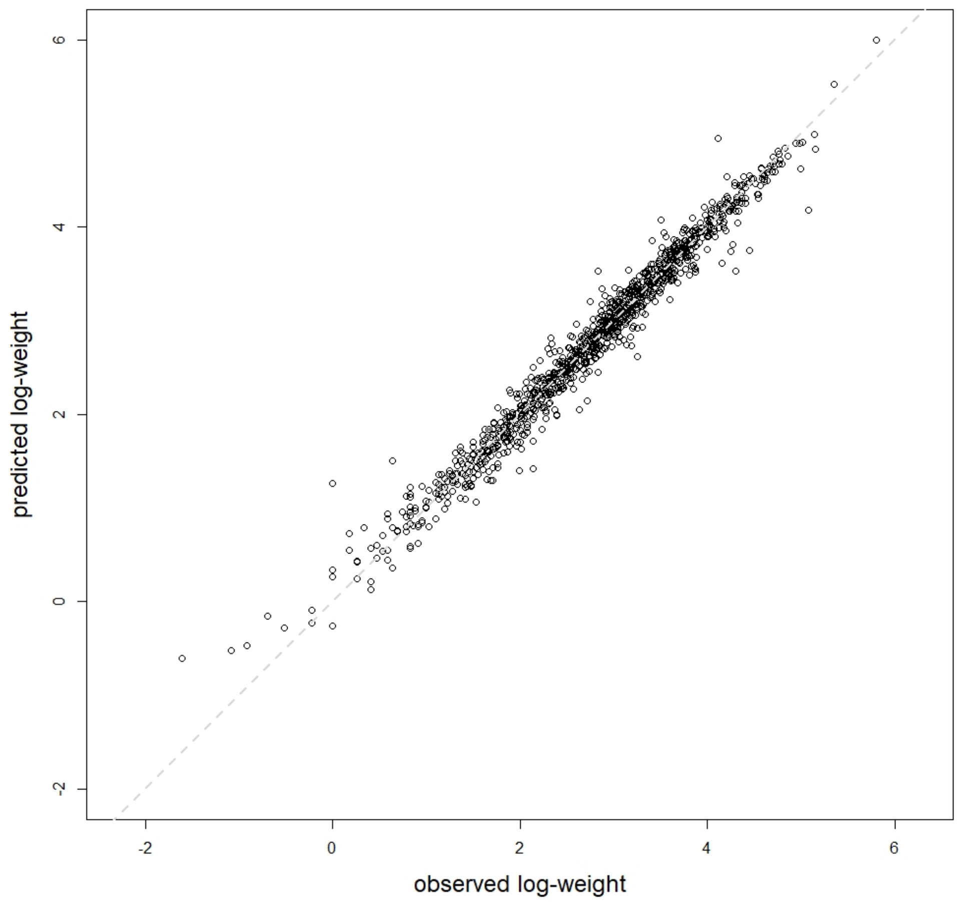

Fig. S1 Relation between the actual (observed) value of fruitbody weight and the value predicted by the partial least squares regression for 2017-2018 data (values log-transformed, n = 1047).

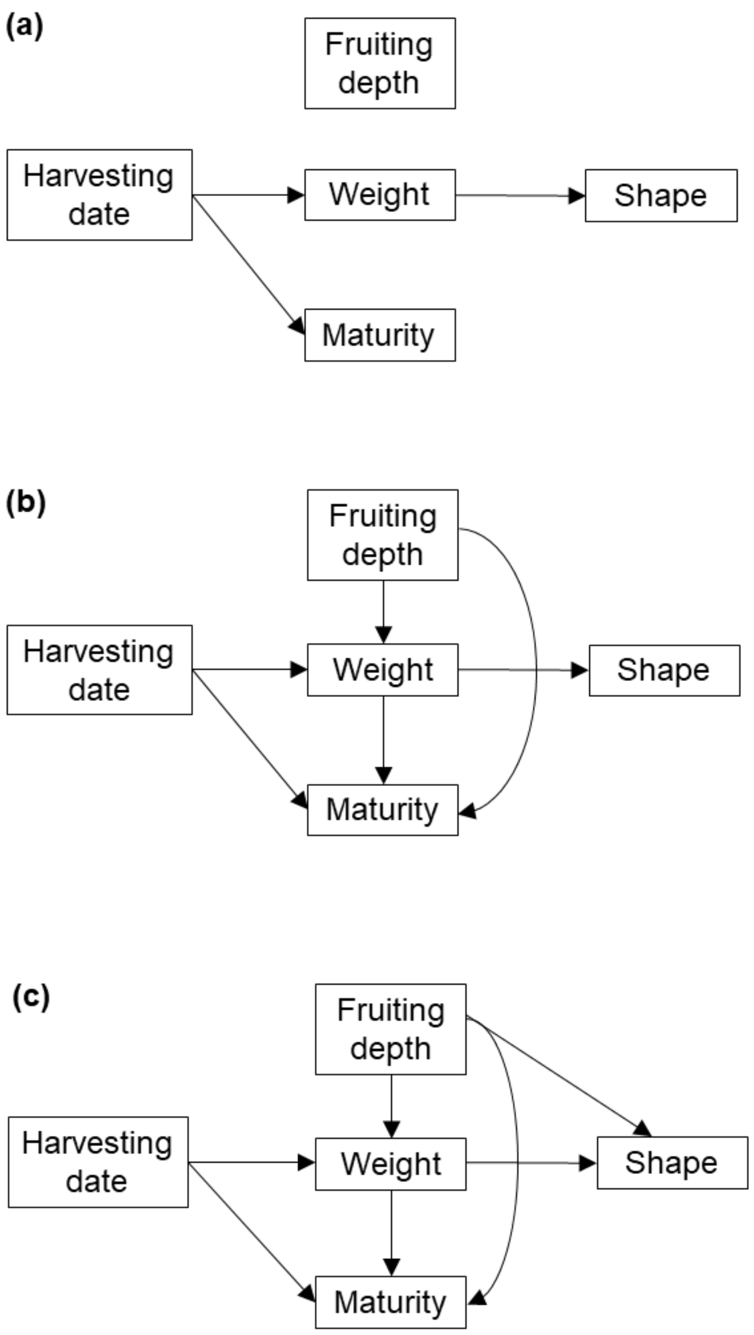

Figure S2 The three alternative directed acyclic graphs tested for single fruitbodies (corresponding to models A-C in Table 2). For simplicity, the error variables and the unspecified links are not shown.

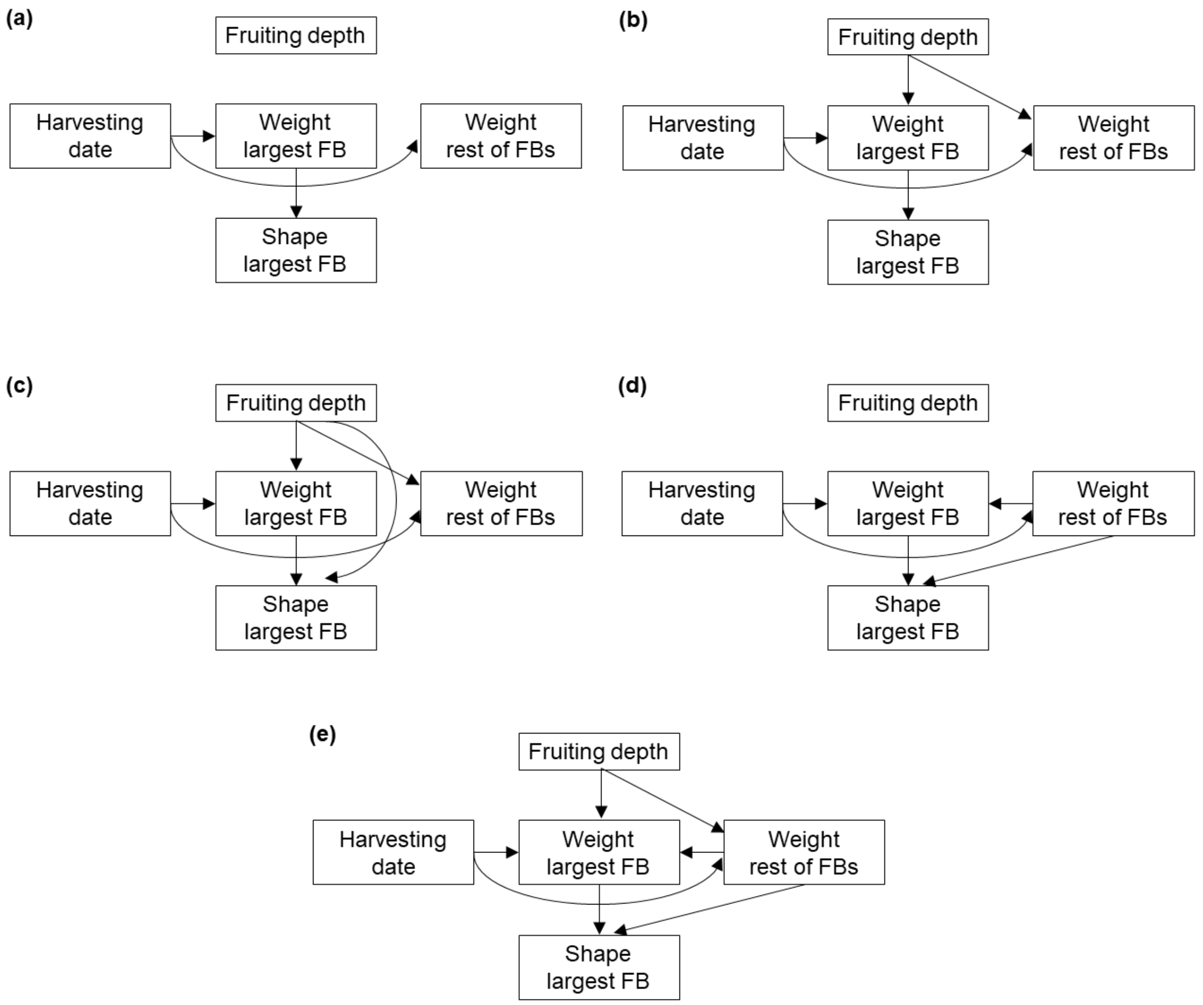

Figure S3 The five alternative directed acyclic graphs tested for fruitbody clusters (corresponding to models A-E in Table 5). For simplicity, the error variables and the unspecified links are not shown. FB: fruitbody.

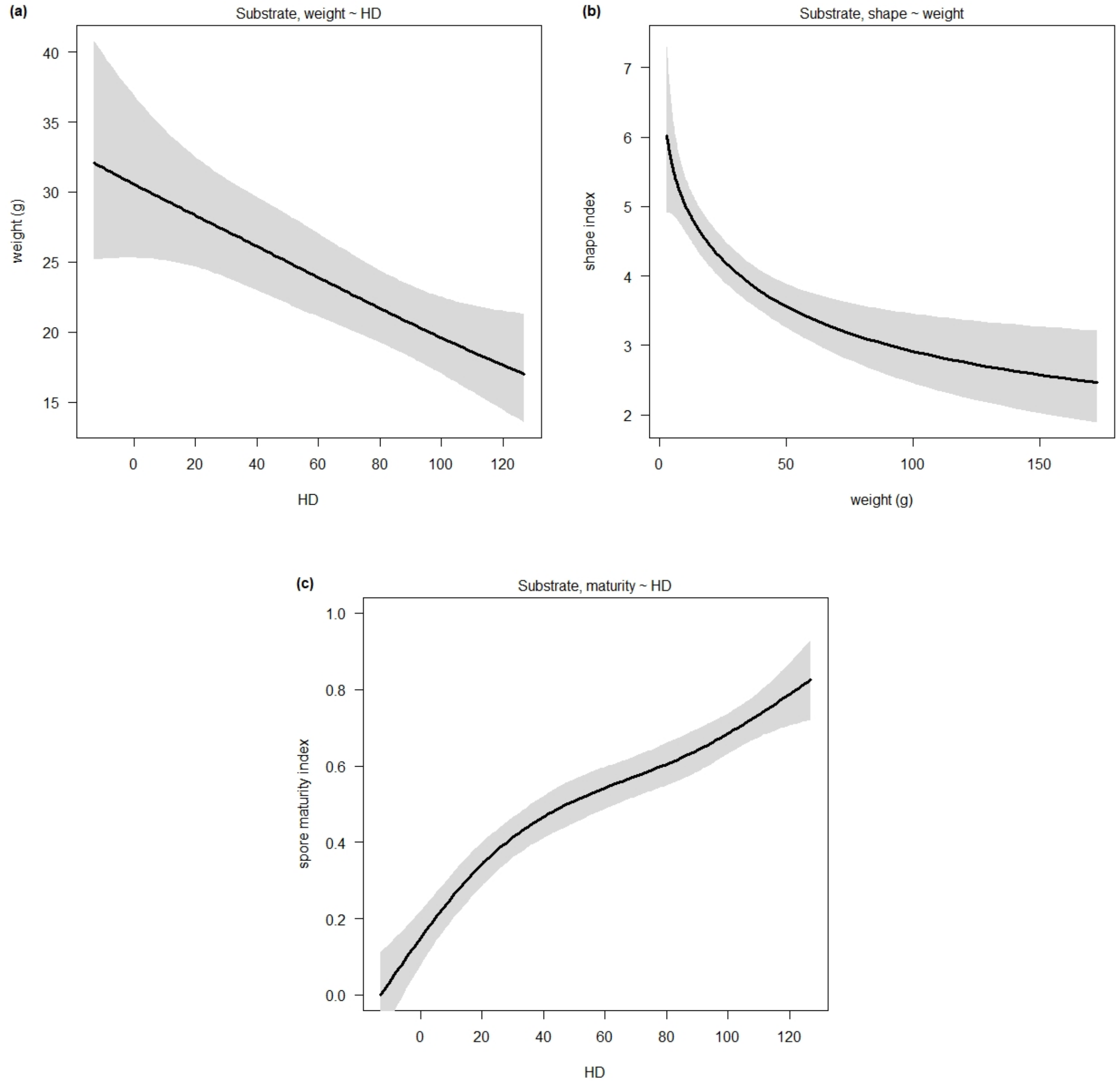

Figure S4 Predicted relations (mean and 95% confidence band) between development characters according to the best-fit model for single fruitbodies in substrate (HD: harvesting date; HD = 0 corresponds to the 15 November and HD = 120 to the 15 March).

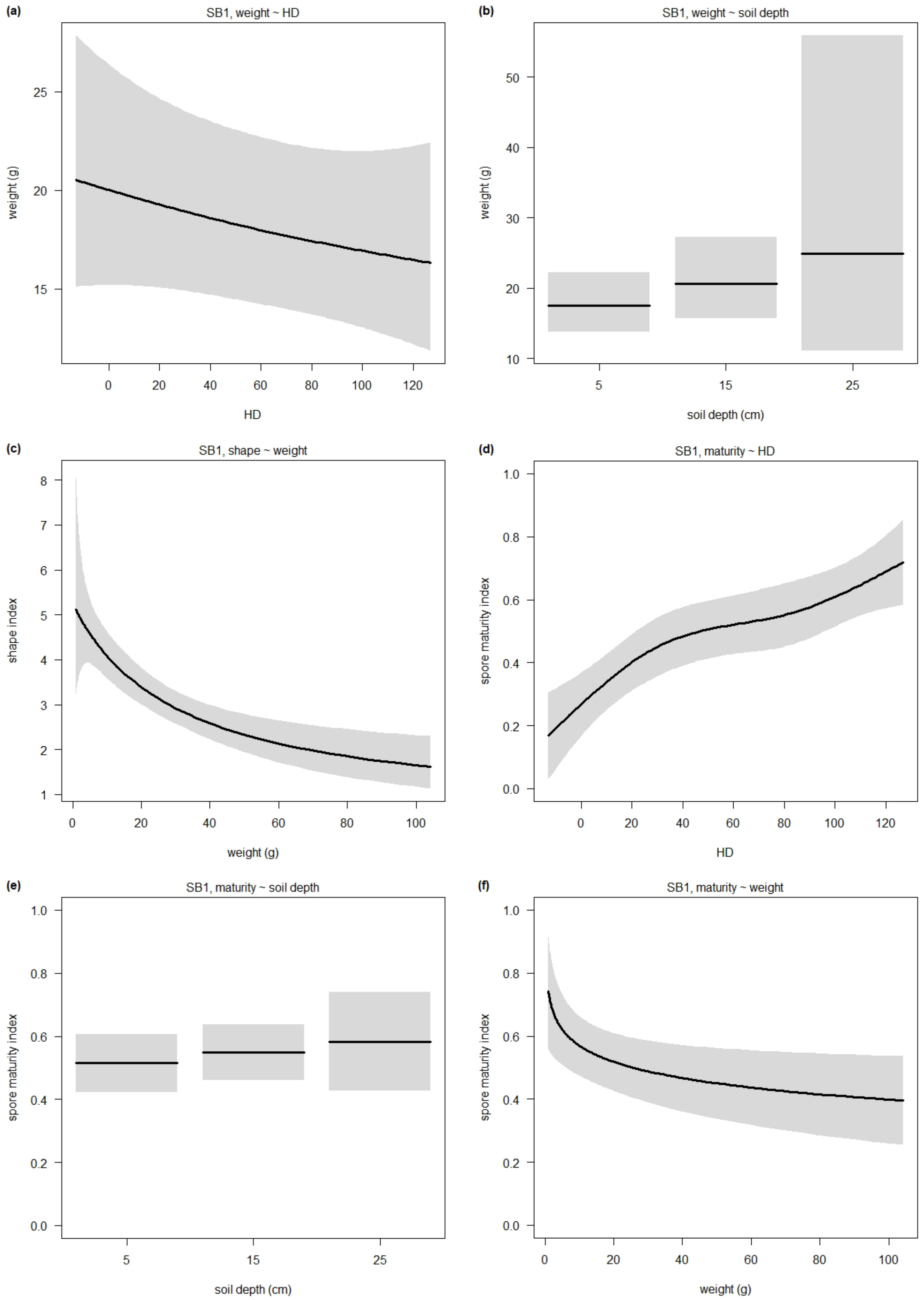

Figure S5 Predicted relations (mean and 95% confidence band) between development characters according to the best-fit model for single fruitbodies in the bulk soil of block 1 (HD: harvesting date; HD = 0 corresponds to the 15 November and HD = 120 to the 15 March).

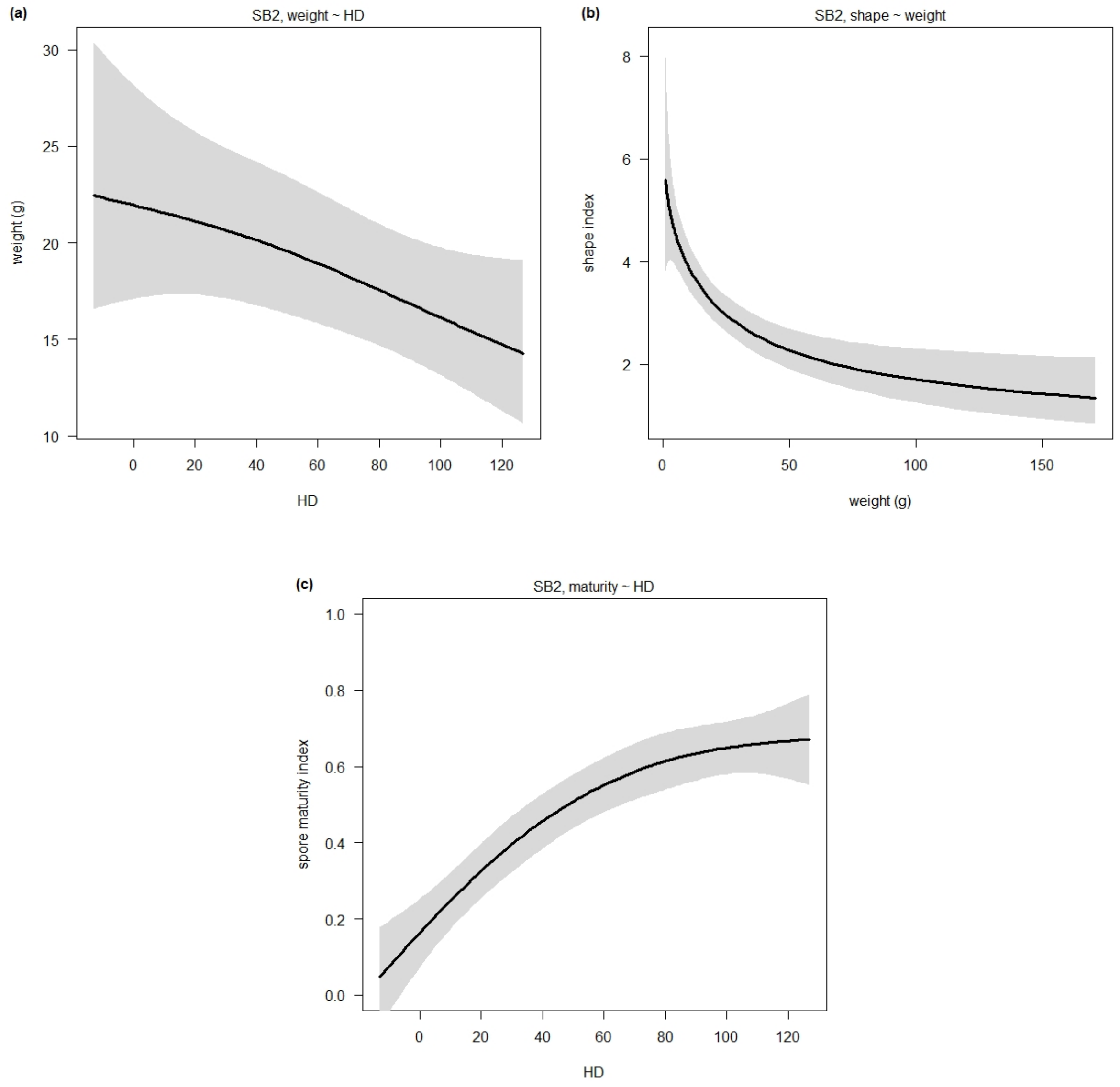

Figure S6 Predicted relations (mean and 95% confidence band) between development characters according to the best-fit model for single fruitbodies in the bulk soil of block 2 (HD: harvesting date; HD = 0 corresponds to the 15 November and HD = 120 to the 15 March).

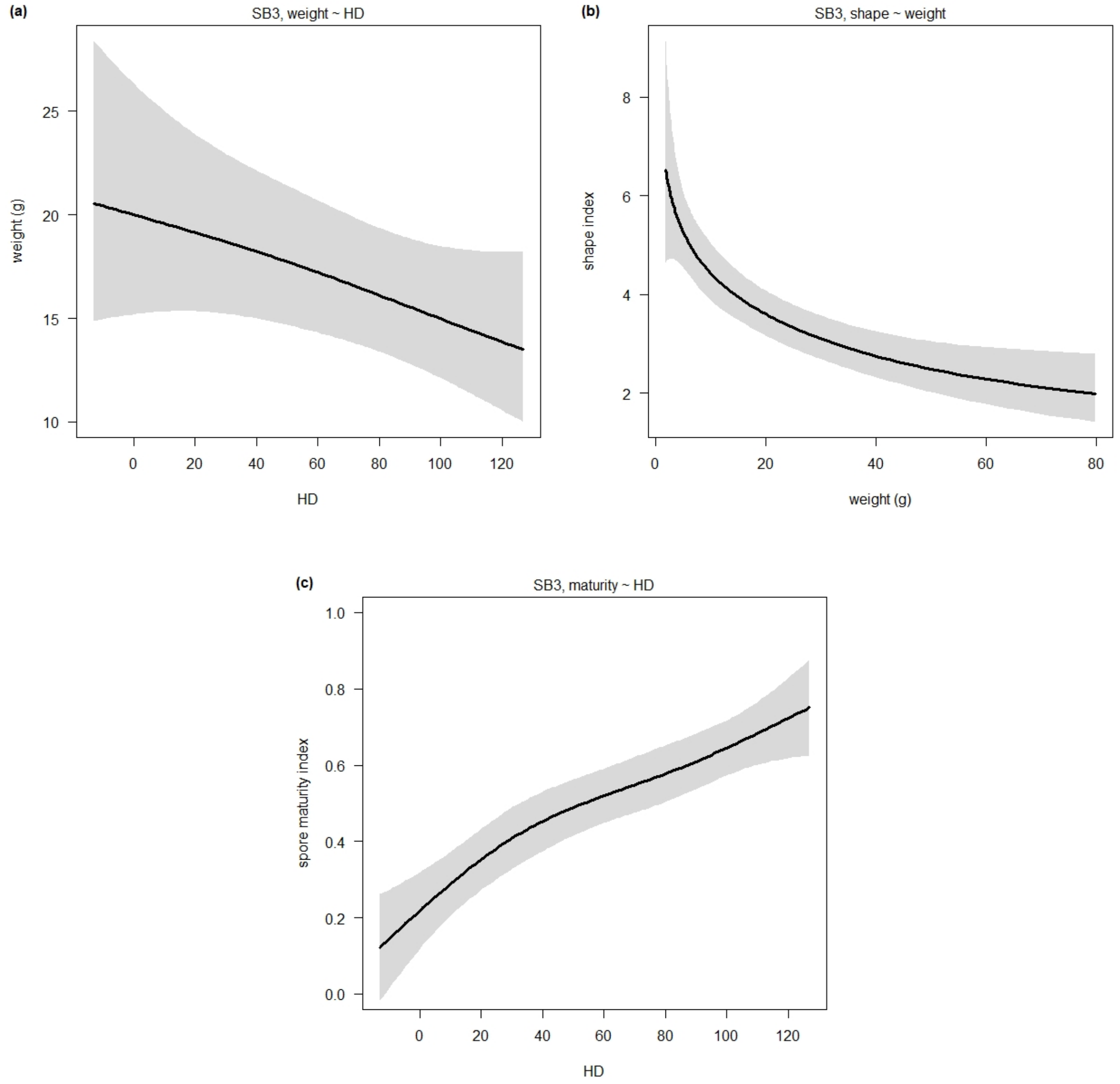

Figure S7 Predicted relations (mean and 95% confidence band) between development characters according to the best-fit model for single fruitbodies in the bulk soil of block 3 (HD: harvesting date; HD = 0 corresponds to the 15 November and HD = 120 to the 15 March).

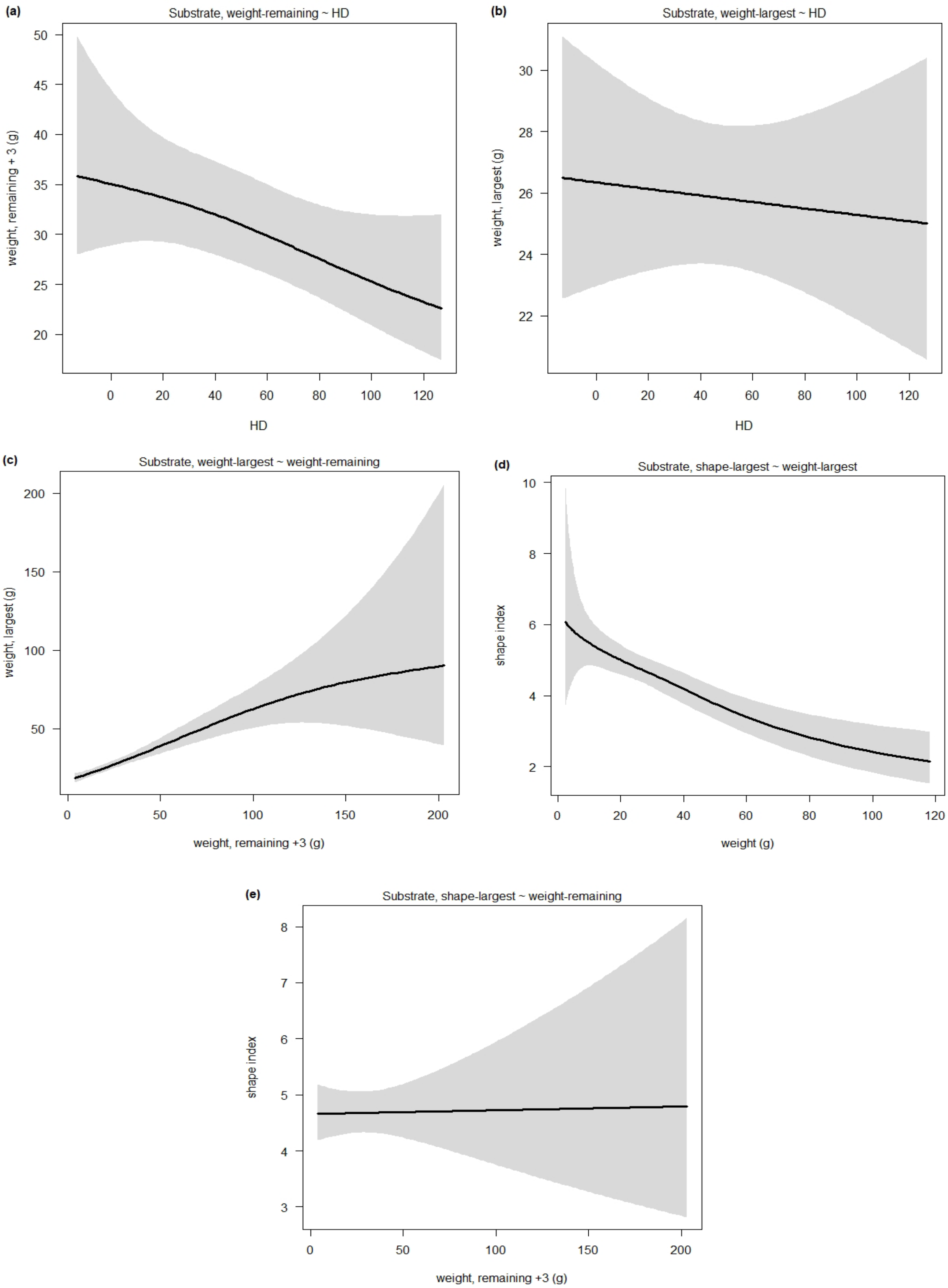

Figure S8 Predicted relations (mean and 95% confidence band) between development characters according to the best-fit model for fruitbody clusters in substrate (HD: harvesting date; HD = 0 corresponds to the 15 November and HD = 120 to the 15 March).

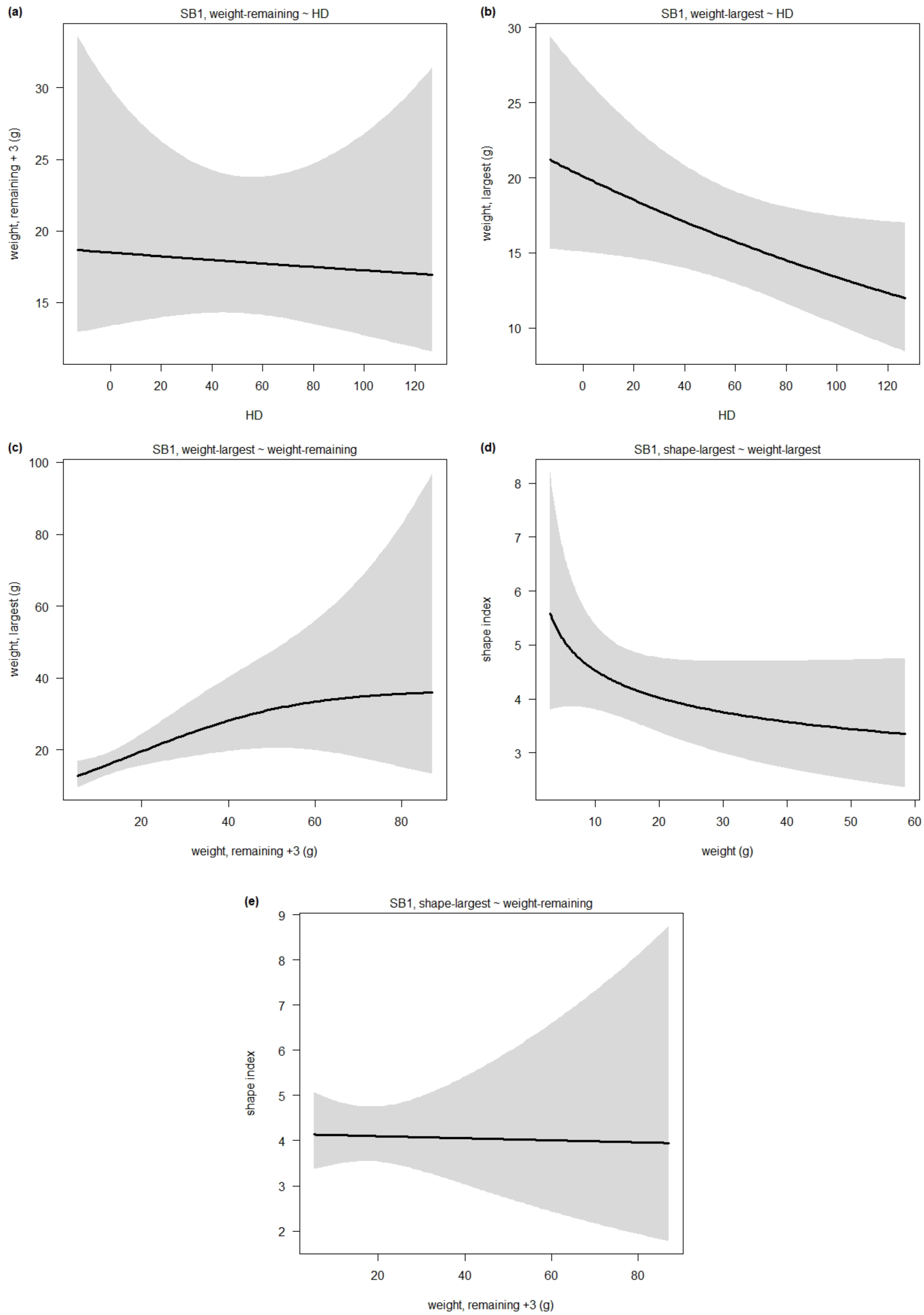

Figure S9 Predicted relations (mean and 95% confidence band) between development characters according to the best-fit model for fruitbody clusters in the bulk soil of block 1 (HD: harvesting date; HD = 0 corresponds to the 15 November and HD = 120 to the 15 March).

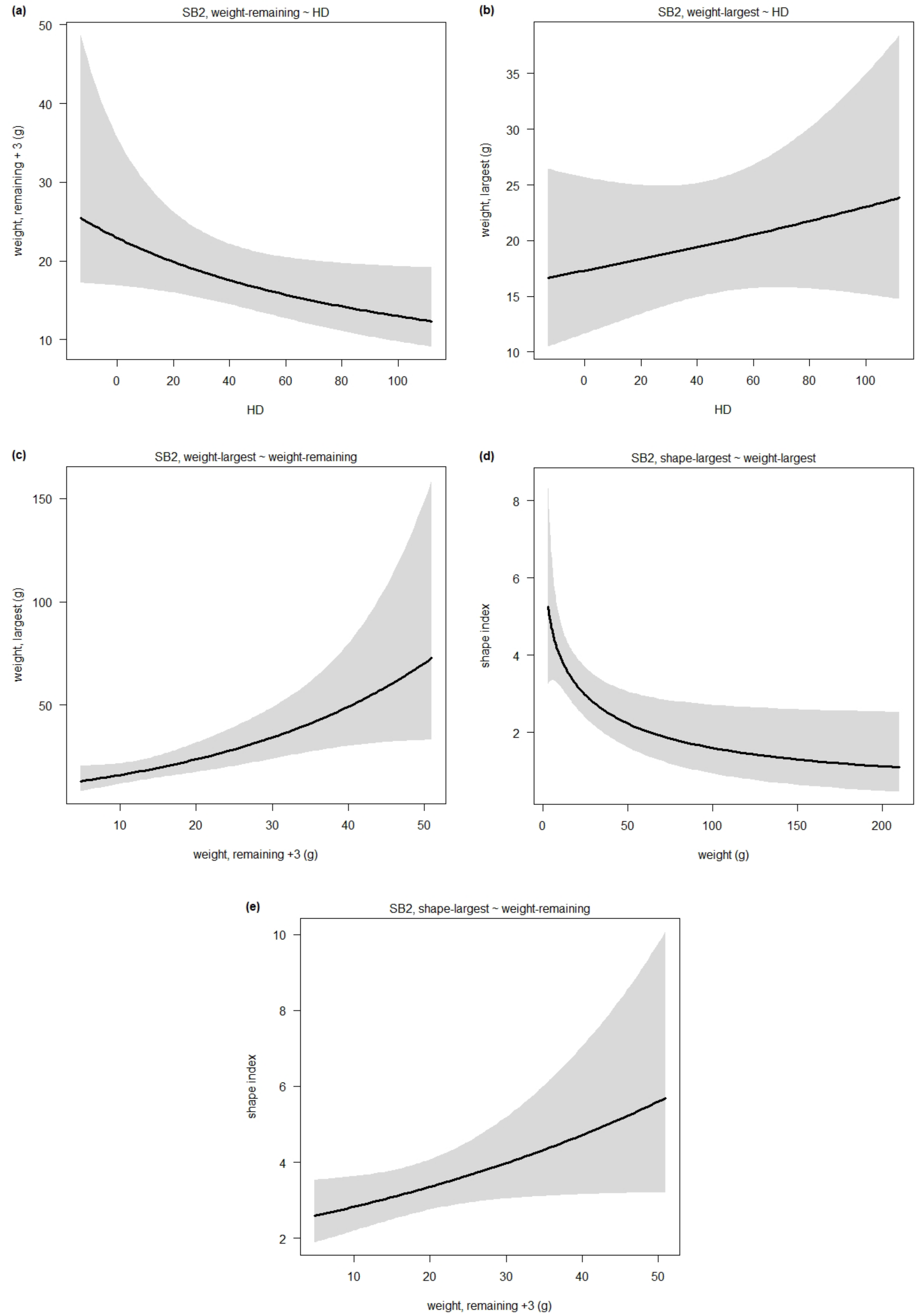

Figure S10 Predicted relations (mean and 95% confidence band) between development characters according to the best-fit model for fruitbody clusters in the bulk soil of block 2 (HD: harvesting date; HD = 0 corresponds to the 15 November and HD = 120 to the 15 March).

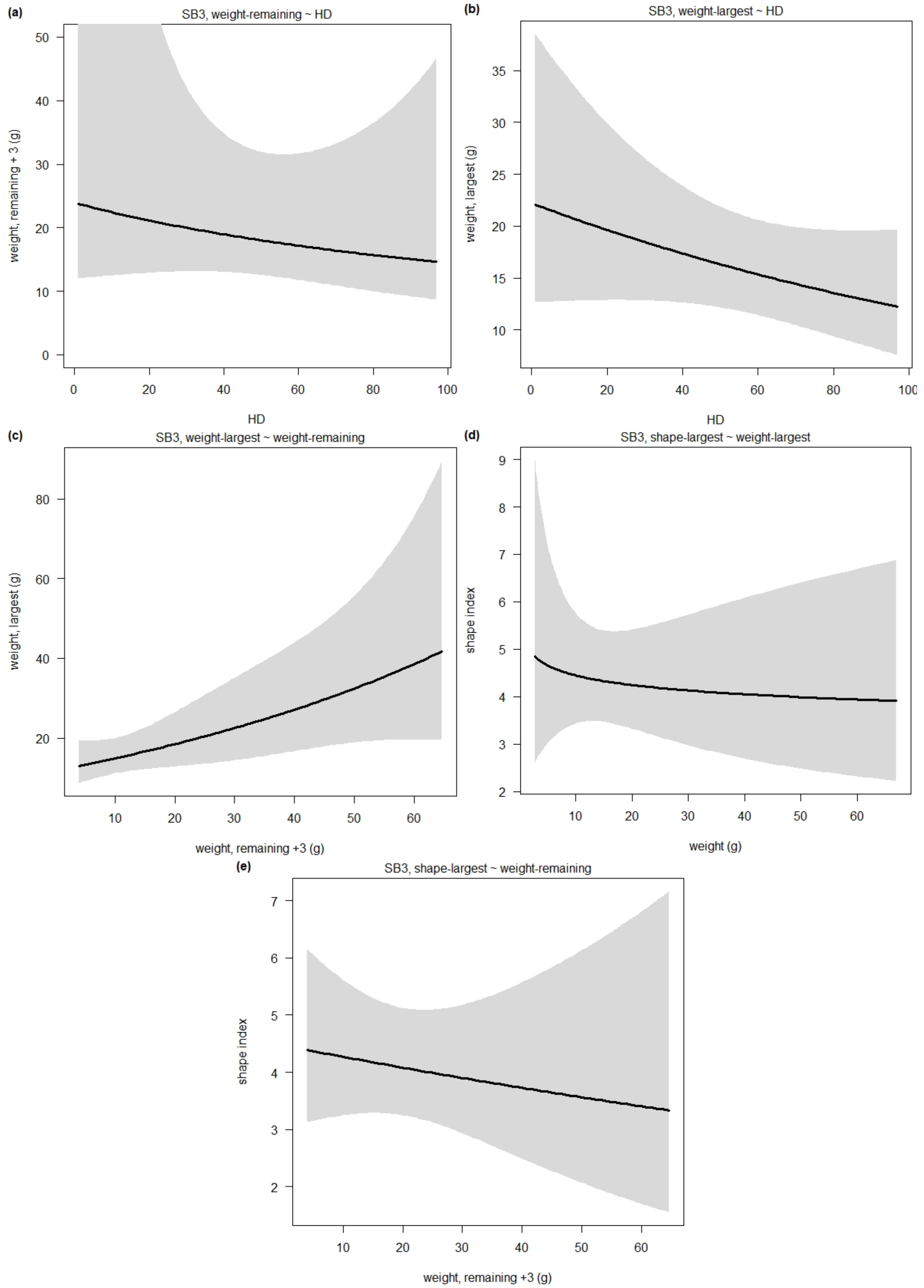

Figure S11 Predicted relations (mean and 95% confidence band) between development characters according to the best-fit model for fruitbody clusters in the bulk soil of block 3 (HD: harvesting date; HD = 0 corresponds to the 15 November and HD = 120 to the 15 March).

Table S6 Descriptive statistics for the development characters of single fruitbodies (SD: standard deviation, N: sample size). Data are disaggregated for the peat-based substrate of the study site and for the bulk soil of each block (BS1, BS2 and BS3).

	Substrate	BS1	BS2	BS3
Weight (g)				
Mean (SD)	32.5 (27.4)	27.8 (22.6)	27.8 (28.1)	23.6 (18.5)
Range	2.8, 173	1.0, 104	1.2, 171	1.9, 80
N	252	114	135	103
Shape index				
Mean (SD)	4.3 (1.8)	3.3 (1.8)	3.3 (1.9)	3.8 (2.0)
Range	0, 8	0, 8	0, 8	0, 8
N	252	114	135	103
Spore maturity				
Mean (SD)	0.50 (0.33)	0.53 (0.33)	0.47 (0.34)	0.50 (0.32)
Range	0, 0.96	0, 0.98	0, 0.92	0, 0.98
N	237	110	129	100
Fruiting depth (absolute and relative frequency)				
0-10 cm	81 (32%)	65 (57%)	69 (51%)	63 (61%)
10-20 cm	152 (60%)	44 (39%)	62 (46%)	40 (39%)
20-30 cm	18 (7%)	5 (4%)	4 (3%)	0
30-40 cm	1 (<1%)	0	0	0

Table S7 Descriptive statistics for the development characters of the fruitbody clusters (SD: standard deviation, N: sample size). Data are disaggregated for the peat-based substrate of the study site and for the bulk soil of each block (BS1, BS2 and BS3).

	Substrate	BS1	BS2	BS3
Weight of the largest fruitbody (g)				
Mean (SD)	34.7 (24.4)	21.9 (13.5)	32.0 (35.0)	21.9 (14.0)
Range	2.6, 118	2.9, 58.5	3.3, 210	2.8, 67
N	203	45	40	20
Shape index for the largest fruitbody				
Mean (SD)	4.5 (1.8)	4.1 (1.7)	3.3 (1.8)	4.2 (1.9)
Range	0, 8	1, 8	0, 7	1, 7
N	203	45	40	20
Weight of the remaining fruitbodies (g)				
Mean (SD)	27.9 (27.9)	14.8 (15.0)	14.6 (11.9)	15.1 (17.9)
Range	1.0, 200	2.3, 84	1.8, 48	1.0, 62
N	203	45	40	
Fruiting depth (absolute and relative frequency)				
0-10 cm	22 (12%)	22 (49%)	18 (60%)	8 (40%)
10-20 cm	135 (77%)	23 (51%)	11 (37%)	12 (60%)
20-30 cm	17 (10%)	0	1 (3%)	0
30-40 cm	2 (1%)	0	0	0
